# Level of COVID-19 fear in cancer patients

**DOI:** 10.1186/s43045-022-00181-5

**Published:** 2022-01-28

**Authors:** Atike Pınar Erdoğan, Ferhat Ekinci, Ömer Acar, Gamze Göksel

**Affiliations:** Department of Internal Medicine, Divison Of Medical Oncology, Manisa Celal Bayar University Faculty of Medicine, Manisa, Turkey

**Keywords:** Cancer, Coronavirus disease 2019 (COVID-19), Pandemic, Fear, Psychology

## Abstract

**Background:**

Cancer patients are in the high-risk group of getting COVID-19 infection and experiencing a severe course. Anxiety of cancer patients about how they pass this pandemic process and how changes in the health system would influence their treatment has increased together with the COVID-19 pandemic. Influence of COVID-19 on psychology of cancer patients is also a subject needed to be investigated as well as its course and prognosis. Thus, it is aimed to measure fear levels of cancer patients by a validated scale. Patients accepting to fill in the validated Fear of COVID-19 (FCV-19S) scale were included in our study. Higher scores obtained from the scale means high level of COVID-19 fear was experienced.

**Results:**

A total of 66.8% of 486 patients expressed that they are very afraid of coronavirus, and 66.3% expressed that they fear from losing their lives due to coronavirus. The level of fear in the patient group having adjuvant therapy has been found statistically to be significantly higher compared with groups having neoadjuvant and metastatic/palliative therapy (*p*: 0.004).

**Conclusions:**

Because the increase of level of fear may lead to vital outcomes such as weakening of immune system, disturbance of treatment compliance, and worsening of prognosis, a psychological approach to cancer patients is compulsory in order to prevent fear of COVID-19 infection.

## Background

COVID-19 pandemic has affected the health system throughout the world, and cancer patients are one of the specific groups in this system. Fear is a characteristic feature differentiating contagious disease from others. This fear may also bring other psychosocial difficulties as it is associated with the rate of transmission, morbidity, and mortality of the invisible factor, as well as cause stigma and isolation [1].

Although researches about psychosocial influences of infectious epidemics appearing in different regions of the world in the past are few, they may guide us in this pandemic process. Depression, anxiety, panic attacks, psychomotor agitation, psychotic manifestations, delirium, and thoughts of suicide have been determined in China during Severe Acute Respiratory Syndrome (SARS) [2]. Psychiatric signs due to SARS have been seen in 22.9% of people applying to the primary (first step) health system in Singapore during SARS, and signs of post-traumatic stress syndrome have been observed in a ratio of 25.8%. Psychiatric signs have been observed more especially in young people and those thinking that they were contagious [3]. Increase of fear level interrupts reasonable decision-making and may lead to lethal consequences such as rejection of oncological therapies in a special population such as cancer patients. Besides stress caused by situations such as facing death with having the diagnosis of cancer, social interactions and changes in body image, coincidence of follow-up, and treatment processes with COVID-19 pandemic makes these patients anxious. Cancer patients experience a period that they become very anxious due to reasons such as other concomitant diseases, taking immunosuppressive treatment, increased risk of infection, postponing surgical interventions, switching health service personnel to other areas or absence of health provider due to becoming ill, not being able to use systems such as telemedicine exclusively. Anxiety of cancer patients increased together with COVID-19 pandemic towards how they will cope with this pandemic process and how changes in the health system due to this infection would influence their therapies [4]. Main problems faced by cancer patients due to the pandemic can be summarized as the possibility of catching disease upon going to and returning from the hospital, difficulty of accessing treatments because of travel restrictions, change of treatment units because of conversion of some hospitals into pandemic hospitals.

More severe signs and complications have been seen in cancer patients with diagnosis of COVID-19 compared with ones not having diagnosis of cancer [5]. While their anxiety related to the course of cancer and treatment was high before COVID-19, anxiety level of patients increases due to the information that the virus influences worse especially the elderly, those with lung cancer, and those with impaired immune system. They experience a challenging period for the psychological aspect by remaining between the requirement for taking life-saving treatments on the one hand and being subjected to risk of contagion on the other hand. It is known that fear and anxiety decrease quality of life and negatively affect the compliance for cancer treatment in cancer patients [6].

While knowledge about the diagnosis process of cancer patients during the COVID-19 pandemic and about how the treatment needs to be planned, there is no sufficient data about psychological problems experienced/to be experienced by cancer patients during pandemic period. Thus, evaluation of fear of COVID-19 in cancer patients by the FCV-19S (Fear of COVID-19), which is a brief, easily understood, easy-to-apply scale used in too many different populations, has come to the agenda now.

It has been aimed in this study that the level of COVID-19 fear of patients who are currently taking and who had completed treatment from our clinic would be evaluated by a validated scale and that its correlation with the type, duration, and other demographic characteristics of cancer disease would be investigated.

## Methods

Patients who presented to the outpatient unit of Manisa Celal Bayar University Medical Oncology Clinic were invited to participate in the survey. Patients who accepted to fill in the Fear of COVID-19 (FCV-19S) scale and sign the informed consent form were included in our study. Demographic and clinical information such as age, gender, type of cancer, age of application, disease stage, and treatments received have been obtained from file records. The FCV-19S scale designed by Ahorsu et al. and validated by many studies was applied to patients [7] (Appendix). The Turkish language form of the scale adapted by Bakioğlu et al. was used [8]. Scores to be obtained from this scale, in which 7 expressions associated with COVID-19 fear were included, where a 5-unit (quinary) Likert (1 (I definitely disagree) to 5 (I definitely agree)) grading was used, range between 7 and 35. Higher scores obtained from the scale means high level of COVID-19 fear was experienced.

Sample size was estimated based on a hypothetical prevalence of fear (considering 50% of prevalence) with a 5% margin of error to be tolerated at the 95% level of confidence, 80% power of the test, and 95% response rate. On this basis, a total sample size of 461 was required.

While findings acquired in the study were evaluated, SPSS (Statistical Package for Social Sciences) for Windows 16.0 program was used for statistical analyses. Compliance of parameters to distribution has been evaluated by the *Kolmogorov–Smirnov* test while study data were evaluated. Descriptive statistics have been prepared as to include frequency (*n*), average, standard deviation, minimum, median, and maximum values. Frequency and percentages have been given for categorical variables. The *Kruskal–Wallis* test was used for comparing parameters not showing normal distribution in quantitative data and *Mann–Whitney U* test was used for comparing two groups. *Spearman’s rho* correlation test analysis was used for comparing correlation between quantitative data. Results were evaluated at 95% confidence interval and significance at *p* < 0.05 level.

## Results

Of the 486 patients included in the study, 324 were females (66.7%) and 162 were males (33.3%). The median age was 55.8 (range 18–85) and 131 patients (27.0%) were above 65 years old. Majority of patients (44.2%) has had the diagnosis of breast cancer. While 273 patients (56.2%) had early-stage disease, 164 patients (33.7%) were at a metastatic stage and 49 patients (10.1%) at a local-advanced stage. The number of patients having active cytotoxic chemotherapy at the time of study was 146 (30%).

When responses given according to articles of FCV-19S were examined separately, 66.8% of patients have expressed that they fear very much from coronavirus, and 66.3% have expressed that they fear from losing their lives due to coronavirus (Table 1).

**Table 1:** Distribution of answers according to articles of Fear of COVID-19 (FCV-19S) scale

Substance	Response	Sample size	Percentage
**I am most afraid of coronavirus-19.**	Don’t agree at all	53	10.9
	Agree a little	72	14.8
	Hesitant	36	7.4
	Mostly agree	109	**22.4**
	Agree completely	216	**44.4**
**It makes me uncomfortable to think about coronavirus-19.**	Don’t agree at all	39	8.0
	Agree a little	83	17.1
	Hesitant	47	9.7
	Mostly agree	112	23.0
	Agree completely	205	42.2
**My hands become clammy when I think about coronavirus-19.**	Don’t agree at all	163	33.5
	Agree a little	104	21.4
	Hesitant	39	8.0
	Mostly agree	71	14.6
	Agree completely	109	22.4
**I am afraid of losing my life because of coronavirus-19.**	Don’t agree at all	62	12.8
	Agree a little	66	13.6
	Hesitant	36	7.4
	Mostly agree	137	**28.2**
	Agree completely	185	**38.1**
**When watching news and stories about coronavirus-19 on social media, I become nervous or anxious.**	Don’t agree at all	65	13.4
	Agree a little	76	15.6
	Hesitant	31	6.4
	Mostly agree	141	29.0
	Agree completely	173	35.6
**I cannot sleep because I’m worrying about getting coronavirus-19.**	Don’t agree at all	199	40.9
	Agree a little	75	15.4
	Hesitant	32	6.6
	Mostly agree	67	13.8
	Agree completely	113	23.3
**My heart races or palpitates when I think about getting coronavirus-19.**	Don’t agree at all	181	37.2
	Agree a little	98	20.2
	Hesitant	31	6.4
	Mostly agree	61	12.6
	Agree completely	115	23.7

Any statistically significant difference has not been found between median scale scores of COVID-19 fear level according to age, gender, age of diagnosis, diagnostic groups, history of chemotherapy, history of radiotherapy, status of currently having chemotherapy, being a control patient, and disease stage (*p* > 0.05) (Table 2).

**Table 2:** General demographic and clinical characteristics of patients

	Sample size	FCV-19S score	***p*** value
**Age groups**			
Under 50 years old	139	22.27 ± 8.09	**0.316**
50–65 years old	216	22.35 ± 8.34	
Above 65 years old	131	23.78 ± 9.47	
**Gender**			
Male	162	21.71 ± 9.22	**0.085**
Female	324	23.21 ± 23.21	
**Tumor type**			
Breast	215	23.45 ± 8.23	**0.435**
Lung	33	21.48 ± 9.92	
Gastrointestinal system	113	22.37 ± 8.76	
Urogenital system	73	22.73 ± 8.35	
Head–neck	19	19.74 ± 8.83	
Other	33	21.97 ± 9.36	
**Disease stage**			
Early	273	23.61 ± 8.3	**0.278**
Local advanced	49	22.63 ± 7.75	
Metastatic	164	21.24 ± 9.14	
**Control patient**			
No	368	22.55 ± 8.66	**0.448**
Yes	118	23.21 ± 8.41	
**Does he/she take active cytotoxic KT?**			
No	340	22.85 ± 8.59	**0.617**
Yes	146	22.38 ± 8.63	

When it is examined according to the purpose of having treatment, fear level in patients having adjuvant therapy has been found to be statistically significantly higher compared with groups having neoadjuvant and metastatic/palliative therapy (*p*: 0.004) (Table 3).

**Table 3:** Evaluation of scores of COVID-19 (FCV-19S) fear scale according to purpose of treatment

Aim of treatment	Sample size	Mean	Standard deviation	Median	Lowest	Highest	***p***
Adjuvant	169	24.15	8.13	23.00	7.00	35.00	**0.004**
Neoadjuvant	29	23.45	7.33	23.00	11.00	35.00	
Metastatic-palliative	136	20.91	9.22	20.00	7.00	35.00	

Targeted therapies being taken by patients have been examined by dividing them into subgroups as hormonotherapy, immunotherapy, tyrosine kinase inhibitor, and monoclonal antibody. Some patients have combination therapies. Fear level in the group having hormonotherapy was found to be statistically significantly higher than other subgroups (*p*: 0.006) (Table 4).

**Table 4:** Evaluation of scores of Fear of COVID-19 (FCV-19S) scale according to groups having targeted therapies

Ones taking targeted therapies	Sample size	Mean	Standard deviation	Median	Lowest	Highest	***p***
Hormonotherapy	124	24.28	7.91	23.00	7.00	35.00	**0.006**
Immunotherapy	11	17.45	9.23	14.00	7.00	35.00	
Tyrosine kinase inh	31	22.06	9.76	23.00	7.00	35.00	
Monoclonal antibody	57	20.49	9.23	19.00	7.00	35.00	

**Fig. 1:**
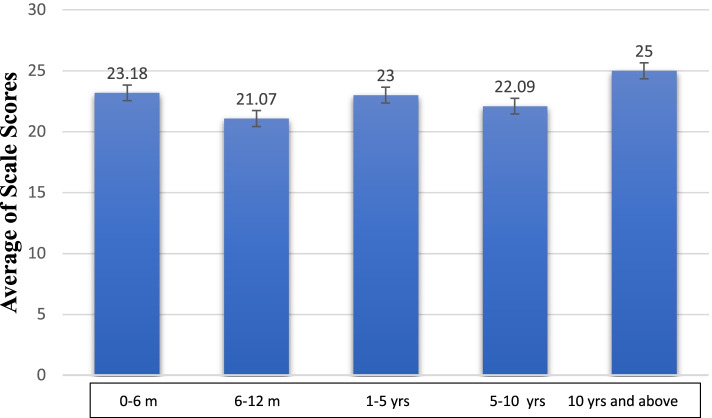
Evaluation of FCV-19S scores according to time from diagnosis of cancer until the time of scale application

When we grouped and examined patients according to the duration from the diagnosis of cancer until the time they answered the FCV-19S scale, it has been observed that the fear level in patients under follow-up for more than 10 years was higher numerically; but this difference has not reached to statistically significant value (*p*: 0.417) (Fig. 1).

**Fig. 2:**
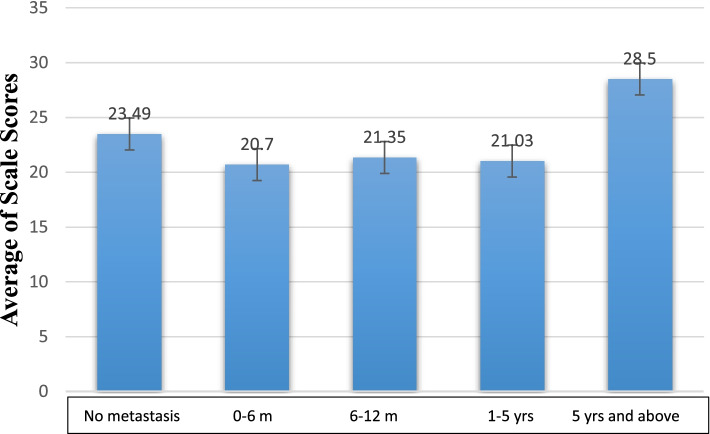
Evaluation of FCV-19S scores from time of metastasis until the time of scale application

When we examined patients by grouping them according to whether a metastasis is present or not and according to time from the date of metastasis development until the time of answering the FCV-19S scale, median fear level in non-metastatic patients and in patients having a metastatic disease for more than 5 years has been found to be higher numerically; but this difference has not reached to a statistically significant value (23.49 and 28.5, respectively; *p*: 0.350) (Fig. 2).

## Discussion

While COVID-19 pandemic takes place as the first pandemic of the 21st century in the history of medicine, it has also caused a lot of changes within the health system unavoidably. It is not difficult to guess that the anxiety of cancer patients has increased about how they pass this pandemic period and how changes in the health system due to this infection would influence their therapies together with COVID-19 pandemic [9]. While their anxiety was high related to the course of cancer and treatment before COVID-19, anxiety level of patients and their relatives increases now because of the information that the virus influences badly especially the people with advanced age, those having lung cancer, and those with impaired immune system [10].

Differently from the general population, it is known that fear and anxiety disorders in cancer patients are not associated with age or gender [11]. In our study, while the median score of the fear scale of male patients was 21.7, it was found to be 23.2 in females and mildly higher than males in numerical terms; however, this difference has not expressed any statistical significance (*p*: 0.085). In another study using the FCV-19S scale in cancer patients, the fear level has been found to be higher in females compared with males (22.2 vs. 17.8, *p*< 0.05) [12].

While the fear score of 131 patients in over-65 years old group that is considered to be at risk for COVID-19 according to data in the literature was 23.7, the score of 355 patients at 65 years old and below has been determined as 22.3 (*p*: 0.316) [13]. Again, in a study where persons at 60 years old and above were included, it has been seen that fear scores of elderly who feel themselves isolated from others and whose close friends and family were diagnosed COVID-19 were higher [14]. It appears that older adults isolated due to immunosuppression related to cancer treatment before the pandemic required closer follow-up for their psychosocial aspect during the period of the COVID-19 pandemic.

There are studies in the literature indicating that depression and anxiety levels are different according to the type of cancer. It is thought that patients having lung cancer and gynecological cancers are affected more frequently [15]. In our study group, breast cancer, gastrointestinal system cancers, and urogenital cancers are dominant according to rank of frequency; levels of fear have been found as 23.45, 22.37, and 22.73, respectively. Although the fear level is numerically high in patients with breast cancer, any statistically significant difference has not been observed (*p*: 0.435). Vanni et al. has examined the influence of their anxiety levels on treatment decisions of patients with breast cancer in the COVID-19 course, and they have indicated that fear occurring due to the contagion risk of COVID-19 infection may be a reason for rejecting surgical intervention [16]. This type of data shows that evaluation of the psychological status is also important in the follow-up and treatment process as well as evaluation of tumor in cancer patients.

Cancer patients are prone to infection due to their immunosuppressed condition caused by the disease itself and treatments such as chemotherapy, radiotherapy, and surgery they had. For this reason, cancer patients form a high-risk population for the aspect of COVID-19. According to the knowledge coming from a study including two small and heterogeneous patient groups, it has been reported that 39–54% of cancer patients catching COVID-19 had severe clinical course (treatment in intensive care unit or death) [17]. The outcome of those having cytotoxic treatment 2–4 weeks before development of symptoms of COVID-19 is worse [18]. In our study, any difference has not been determined between fear levels of 146 patients having cytotoxic chemotherapy on the day the scale was used compared with those having other oncological treatments or patients who completed treatment and come for control visits (22.3 and 22.85, respectively). In the study of Sigorski et al., the FCV-19S scale has been used at the second month of the pandemic, and fear level of cancer patients having cytotoxic therapy has been reported as 18.5 which is lower than our population [19]. At the time our study was conducted, the pandemic has completed its 11th month in our country, and the total number of cases has exceeded 2.5 million, and the number of daily new cases was around an average of 7500 [20]. Prolongation of the pandemic process and increase of uncertainties have increased the level of fear, and this may cause the fact that scores have been found higher in our study.

The fear level of 124 patients having hormonal agents which have been considered as one of the safe oncological treatments has been found to be higher compared with patients having immunotherapy, tyrosine kinase inhibitor, and monoclonal antibody during the period of the COVID-19 pandemic, and this difference is statistically significant (*p*: 0.006, scores are 24.2 vs. 17.4, 22, 20.4, respectively). This difference can be explained by the fact that fear level of these patients is higher, most of whom have early-stage disease, because hormonal agents are used for adjuvant purpose in breast cancer.

While the fear scale score of patients not having metastasis was 23.49, score of those having metastatic disease for 1–5 years has been determined to be 21.03. This observation made us think that metastatic cancer patients have vital anxieties of more priority than those with COVID-19 during the struggle with cancer. The higher level of fear, due to COVID-19, of early-stage patients who have expectations for cure may be related to the anxiety that successful result they can obtain from cancer therapy may be interrupted by COVID-19 infection.

Hemmington et al. have demonstrated that there are severe mental health problems in patients with advanced-stage cancer; in our study, while fear scale scores of patients with diagnosis of metastasis for 0–6 months, 6–12 months, and 1–5 years are 20.70, 21.35, and 21.03, respectively, the score of 6 patients having metastatic diseases for more than 5 years has the highest score with an average of 28.5; but further interpretation has not been made because the number of patients is small in this group (*p*: 0.350) [6].

It is known that depression and anxiety are at a high level in the acute period when cancer diagnosis was made, and it would decrease with time [21]. While the score from the fear scale of patients included in the study was 22.09 whose duration from the diagnosis is between 5 and 10 years, a score of 23.18 has been determined from those having the diagnosis within the last 6 months. Although it does not reach to a statistically significant value, it can be said that patients newly diagnosed with cancer may cause an increase of COVID-19 fear level, and perception level of patients for both diseases might be influenced consistently with the literature.

Our study has several limitations. Primarily, findings may not be applied to the general oncological patient population because it was conducted in a tertiary oncology clinic, and participation was based on voluntariness principle. Another point is the use of psychotropic drugs that may influence perception of patients towards expression in the scale has not been recorded. Making an evaluation in a relatively large sample of patients in different age groups and tumor types by a validated scale is the strong side of our study.

Cancer patients were familiar with wearing masks in the community, staying away from crowds, and showing isolation behavior also prior to the COVID-19 pandemic. However, because cancer patients, who take precautions to avoid getting infected for a long time, experienced some kind of “precaution tiredness” with the pandemic process, disturbance of treatment compliance and emergence of mental health problems or exacerbation of existing ones are subjects for question. Psychosocial approaches or psychological evaluations towards the mental health of cancer patients should certainly be made for each patient during the pandemic period. Worsening of the immune system due to increase of anxieties and irritability, lack of motivation, tiredness, fatigue, and signs of depression may appear at this period, and these may influence the treatment process and even the prognosis [22]. Randomized controlled studies on how the patients’ anxiety symptoms related to the fear of COVID-19 affect their general condition, laboratory values, and drug responses are needed.

## Conclusions

In order to prevent fear of COVID-19 infection which takes patients away from life-saving treatments and which causes death, psychological evaluation of cancer patients is compulsory. Researches about influence of COVID-19 on psychology of cancer patients are needed as well as its course and prognosis in cancer patients.

## Data Availability

The datasets used and/or analyzed during the current study are available from the corresponding author on reasonable request**.**

## Appendix

Fear of Coronavirus-19 Scale

1. I am most afraid of coronavirus-19.

2. It makes me uncomfortable to think about coronavirus-19.

3. My hands become clammy when I think about coronavirus-19.

4. I am afraid of losing my life because of coronavirus-19.

5. When watching news and stories about coronavirus-19 on social media, I become nervous or anxious.

6. I cannot sleep because I’m worrying about getting coronavirus-19.

7. My heart races or palpitates when I think about getting coronavirus-19.

The participants indicate their level of agreement with the statements using a five-item Likert-type scale. Answers included “strongly disagree,” “disagree,” “neither agree nor disagree,” “agree,” and “strongly agree”. The minimum score possible for each question is 1, and the maximum is 5. A total score is calculated by adding up each item score (ranging from 7 to 35). The higher the score, the greater the fear of cororonavirus-19.

## Abbreviations

FCV-19S: Fear of COVID-19 scale
SARS: Severe Acute Respiratory Syndrome
